# Incremental Experience in In Vitro Primary Culture of Human Pulmonary Arterial Endothelial Cells Harvested from *Swan-Ganz* Pulmonary Arterial Catheters

**DOI:** 10.3390/cells10113229

**Published:** 2021-11-19

**Authors:** Birger Tielemans, Leanda Stoian, Allard Wagenaar, Mathias Leys, Catharina Belge, Marion Delcroix, Rozenn Quarck

**Affiliations:** 1 Laboratory of Respiratory Diseases & Thoracic Surgery (BREATHE), Department of Chronic Diseases & Metabolism (CHROMETA) & Biomedical MRI, Department of Imaging and Pathology, University of Leuven, 3000 Leuven, Belgium; birger.tielemans@kuleuven.be; 2 Laboratory of Respiratory Diseases & Thoracic Surgery (BREATHE), Department of Chronic Diseases & Metabolism (CHROMETA), University of Leuven, 3000 Leuven, Belgium; leandavengethasamy@yahoo.com (L.S.); allard.wagenaar@kuleuven.be (A.W.); 3 Clinical Department of Respiratory Diseases, University Hospitals, University of Leuven, 3000 Leuven, Belgium; MATHIAS.LEYS@azgroeninge.be; 4 Laboratory of Respiratory Diseases & Thoracic Surgery (BREATHE), Department of Chronic Diseases & Metabolism (CHROMETA), Clinical Department of Respiratory Diseases, University Hospitals, University of Leuven, 3000 Leuven, Belgium; catharina.belge@uzleuven.be (C.B.); marion.delcroix@uzleuven.be (M.D.)

**Keywords:** pulmonary arterial hypertension, endothelial cells, right heart catheterization, diagnosis, cell culture

## Abstract

Pulmonary arterial hypertension (PAH) is a devastating condition affecting the pulmonary microvascular wall and endothelium, resulting in their partial or total obstruction. Despite a combination of expensive vasodilatory therapies, mortality remains high. Personalized therapeutic approaches, based on access to patient material to unravel patient specificities, could move the field forward. An innovative technique involving harvesting pulmonary arterial endothelial cells (PAECs) at the time of diagnosis was recently described. The aim of the present study was to fine-tune the initial technique and to phenotype the evolution of PAECs in vitro subcultures. PAECs were harvested from *Swan-Ganz* pulmonary arterial catheters during routine diagnostic or follow up right heart catheterization. Collected PAECs were phenotyped by flow cytometry and immunofluorescence focusing on endothelial-specific markers. We highlight the ability to harvest patients’ PAECs and to maintain them for up to 7–12 subcultures. By tracking the endothelial phenotype, we observed that PAECs could maintain an endothelial phenotype for several weeks in culture. The present study highlights the unique opportunity to obtain homogeneous subcultures of primary PAECs from patients at diagnosis and follow-up. In addition, it opens promising perspectives regarding tailored precision medicine for patients suffering from rare pulmonary vascular diseases.

## 1. Introduction

Pulmonary arterial hypertension (PAH) is a rare but devastating condition, characterized by a precapillary pulmonary arteriopathy resulting in partial or total obstruction, leading to increased pulmonary vascular resistance (PVR), elevated mean pulmonary arterial pressure (mPAP) and eventually right heart failure. PAH is associated with mutations in the bone morphogenetic protein receptor type II (BMPR2) gene [1], impaired BMPRII signaling, pulmonary vascular remodeling and endothelial dysfunction [2]. Despite the current combination of effective vasodilatory therapies, mortality rates remain high, with a 5-year survival rate of 49–67% [3]. Currently, there is no curative treatment available for PAH, with lung transplantation being the only option for selected patients with severe PAH refractory to current medical therapies [4].

To better understand the onset and progression of PAH, various experimental animal models have been developed [5]. Despite their contribution to the insight of the pathogenesis, they all display limitations to efficiently translating their outcome to the bedside. In vitro alternatives based on pulmonary material from transplanted patients imply major drawbacks, including a limited number of samples and the advanced stage of disease of patients exposed to PAH-specific drugs for long periods. Therefore, innovative methods of accessing patient material are necessary to better understand disease progression and heterogeneity. For instance, transient and stable knock-down of *bmpr2* in human pulmonary vascular cells has been established to investigate the effect of impaired BMPRII signaling on disease progression [6]. Obtaining endothelial colony–forming cells derived from peripheral blood is an elegant technique that allows the generation of induced pluripotent stem cells (iPSC) from PAH patients at diagnosis and from healthy *bmpr2* mutation carriers [7]. The use of iPSC recently uncovered modifiers of BMPRII signaling that protect against PAH in *bmpr2* mutation carriers, underscoring the need to investigate patient-specific material [8]. 

An alternative method to accessing patient material is to isolate pulmonary arterial endothelial cells (PAECs) from the balloon located at the tip of *Swan-Ganz* pulmonary arterial catheters, as initially published several years ago by Pollett et al. [9]. More recently, Ventetuolo et al. moved this research forward by showing that cultures and characterization of these PAECs is feasible [10]. 

In this study, we aim to report our incremental experience in phenotyping PAECs throughout in vitro subcultures.

## 2. Materials and Methods

### 2.1. Isolation of PAECs from Swan-Ganz Pulmonary Arterial Catheters

The distal part of *Swan-Ganz* pulmonary arterial catheters were collected from patients at the time of routine diagnostic or follow-up right heart catheterization performed in patients with idiopathic PAH (IPAH), heritable PAH (HPAH), PAH associated with congenital heart or connective tissue diseases, porto-pulmonary hypertension, pulmonary veno-occlusive disease, chronic thromboembolic pulmonary hypertension and PH associated with lung or heart diseases at the University Hospitals of Leuven between 2015 and 2019. The study protocol was approved by the institutional ethics committee and all participants gave written informed consent.

**Figure 1:**
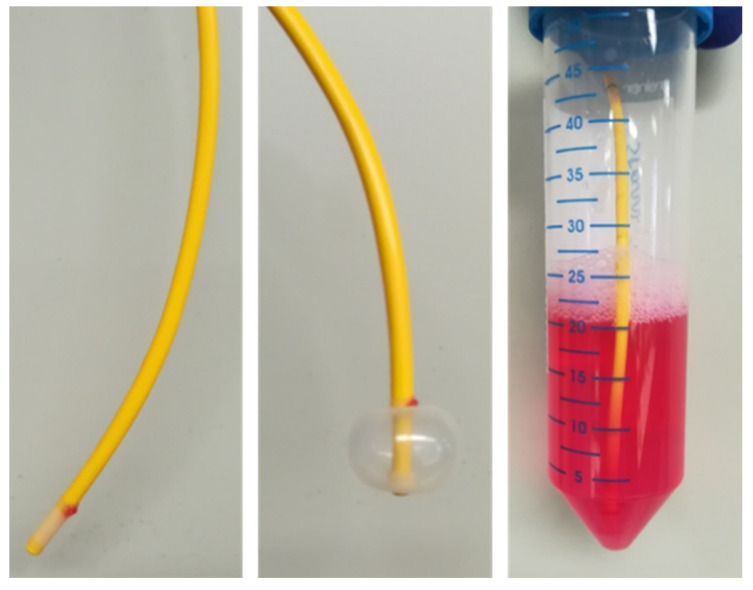
*Swan-Ganz* pulmonary arterial catheter with inflatable balloon at the tip of the catheter is collected in warm (37 °C) basal medium containing 0.2% cell growth supplement and transported from the catheterization unit to the laboratory.

To harvest PAECs from *Swan-Ganz* pulmonary arterial catheters, we adapted and fine-tuned the method initially described by Pollett et al. [9]. The catheter balloon was withdrawn protected within the sheath and immediately placed in previously warmed (37 °C) microvascular endothelial cell basal medium supplemented with 0.2% cell growth supplement (Cell Applications Inc., San Diego, CA, USA), antimycotics (1.25 µg/mL amphotericin B; Thermo Fisher Scientific, Waltham, MA, USA) and antibiotics (100 U/mL penicillin, 100 µg/mL streptomycin; Thermo Fisher Scientific). The balloon was inflated and agitated thoroughly for 2 min. The tip of the catheter was cut, maintained in warm medium and transferred to the laboratory within 10 min for further processing (Figure 1).

Under the laminar flow, the balloon was inflated with air using a 1-mL syringe and transferred into a 0.25% trypsin solution containing 0.91 mM EDTA (Thermo Fisher Scientific). After 2 min, the catheter tip was washed briefly in fresh microvascular endothelial cell growth medium containing 6% cell growth supplement (Cell Applications Inc.), antimycotics and antibiotics, as abovementioned. This medium was mixed with the trypsin solution and centrifuged together with the collection tube at 500× *g* at room temperature for 10 min. To discard red blood cells, the resulting pellets were pooled and incubated in ammonium–chloride–potassium lysis buffer (Thermo Fisher Scientific) for 5 min at room temperature, and were then centrifuged at 500× *g* at room temperature for 10 min, with an excess of phosphate-buffered saline (PBS). The remaining pellet was resuspended in microvascular endothelial cell growth medium, seeded in 2 wells of a 12-well cell culture plate coated with gelatin (2 mg/mL; Sigma-Aldrich, Saint-Louis, MO, USA) and maintained at 37 °C under 5% CO_2_. The cell culture medium was refreshed after five days and then every second day. Cells were maintained up to subculture 7 to 12 until no further subculture was possible, including trypsinization every 4 to 5 days, for 6 to 9 weeks. Subculture is defined as cell trypsinization and consequent transfer to a larger culture surface, with a constant seeding cell density of 12,500 cells per cm^2^. They were retrospectively depicted as early (<3) and advanced subcultures (between 7 and 10).

For long-term storage in liquid N_2_ at −170 °C, cells were expanded, trypsinized and resuspended in fetal bovine serum containing 10% dimethyl sulfoxide (10^6^ cells/vial).

A culture was considered successful if several colonies emerged and expanded within 4 weeks after isolation. A few successful cultures could not be frozen and stored due to bacterial/fungal contamination or inability to sufficiently expand.

### 2.2. Quantitative Phenotyping by Flow Cytometry

Subconfluent PAECs were trypsinized, resuspended in microvascular endothelial cell growth medium and counted using the Countess™ automated cell counter (Thermo Fisher Scientific). One hundred thousand cells were collected for negative control and for staining with allophycocyanin-conjugated anti-CD31 antibody (APC-conjugated CD31; Miltenyi Biotec, Leiden, The Netherlands). Cells were centrifuged at 300× *g* and 4 °C for 10 min, resuspended in PBS containing 1% BSA (PBS-1% BSA) and incubated with APC-conjugated CD31 for 15 min in the dark at 4 °C. Cells were further washed with PBS-1% BSA, fixed in PBS containing 4% paraformaldehyde during 15 min in the dark at 4 °C and resuspended in PBS-1% BSA for further quantitative flow cytometry analysis (Canto HTS, BD Bioscience, Franklin Lakes, NJ, USA). Analysis was standardized with fixed gates used to investigate CD31-APC positive cells as previously described [6]. Forward scatter cell (FSC) density plots were used to evaluate the size of cells and further exclude cell debris. A CD31-APC parameter histogram was used to identify CD31-APC positive cells.

### 2.3. Qualitative Phenotyping of PAECs Using Immunofluorescence

Acetylated low-density lipoprotein (Ac-LDL) displays the unique property of binding to scavenger receptors expressed by endothelial cells and has been previously used to characterize endothelial cells [11,12]. Subconfluent PAECs were seeded onto fibronectin-coated (10 µg/mL; R&D Systems, Abingdon, UK) 4-chamber slides (Thermo Fisher Scientific) at a density of 20,000 cells/chamber and further labeled using 10 µg/mL Ac-LDL coupled with fluorescent DiI (1,1′-dioctadecyl-3,3,3′,3′-tetramethylindocarbocyanine perchlorate, Tebu Bio, Le Perray-en-Yvelines, France) for 4 h at 37 °C and fixed in PBS containing 4% paraformaldehyde. 

In addition, PAECs were phenotyped by immunofluorescence using antibodies against human CD31 (Clone JC70A, Agilent, Santa Clara, CA, USA), von Willebrand factor (vWF, clone F8/86; Agilent) and Vascular Endothelial-Cadherin (VE-Cadherin, clone D87F2; Cell Signaling Tech., Danvers, MA, USA). Potential contamination with myofibroblasts at early subcultures and cell dedifferentiation in advanced late subcultures were investigated by immunostaining using antibodies raised against human alpha smooth muscle actin (α-SMA, clone 1A4; Agilent). Cells were seeded onto fibronectin-coated 4-chamber slides (20,000 cells/chamber). Subconfluent PAECs were fixed in PBS containing 4% paraformaldehyde and permeabilized with 0.2% Triton-X100 (Sigma-Aldrich). Non-specific binding sites were saturated in PBS containing 3% bovine serum albumin (BSA; Sigma-Aldrich) for 1 h at room temperature. Immunolabeling of endothelial-specific markers was performed using primary antibodies diluted in PBS containing 3% BSA for 2 h at room temperature (CD31, 1:25 dilution; vWF, 1:50 dilution; VE-Cadherin, 1:600 dilution; α-SMA, 1:50 dilution). Fluorescent labeling was obtained using secondary antibodies Alexa594 (1:2000 dilution; Thermo Fisher Scientific) goat anti-mouse for CD31, vWF and α-SMA and goat anti-rabbit for VE-cadherin for 1 h at room temperature. For negative control, primary antibodies were omitted. Nuclei were visualized using 4′,6-diamino-2-phenylindole (DAPI, Thermo Fisher Scientific). Slides mounted in FluoroSave (Calbiochem, San Diego, CA, USA) medium were analyzed under an inverted IX80 fluorescence microscope (Olympus, Shinjuku, Japan). To quantify immunofluorescence staining, 3 images from non-overlapping fields on each slide were captured at 40× magnification. After separation of the different channels, red staining was measured and nuclei counted using the ImageJ software and expressed as arbitrary units (AU) per cell. Quantification of cell hypertrophy was performed using immunofluorescent images of CD31 staining, measuring the cell surface area of ten cells in three different fields using the ImageJ software with Java version 1.8.0_231 (ImageJ, National Health Institute, Bethesda, MD, USA). 

### 2.4. Statistical Analysis

Statistical analyses were performed using GraphPad Prism 8.1.2 (GraphPad Software Inc., San Diego, CA, USA). Differences between early and late subcultures were analyzed using a paired *t*-test. Values (demographic and clinical parameters) not normally distributed were log-transformed and expressed as median (min-max); differences between two groups were analyzed using Student’s *t* test. All p values were for 2 sided tests. A value of *p* < 0.05 was considered statistically significant. Values are expressed as mean ± SD.

## 3. Results

### 3.1. Collection of Patient Cells and Patient Characteristics

Between 2015 and 2019, we collected 132 *Swan-Ganz* pulmonary arterial catheters; most of the catheters (69%) were issued from patients with idiopathic PAH, heritable PAH, toxin-drug-induced PAH or chronic thromboembolic pulmonary hypertension (Table 1). Analysis of the patient characteristics indicated no significant difference between successful and unsuccessful procedures (Table 1).

We observed growing cells from 56 procedures, as well as expanded and stored cells from 23 procedures, with significantly improved success rate over time (Table 2). 

### 3.2. Quantitative Phenotyping of PAECs throughout Subcultures

PAECs from four patients were randomly selected; their characteristics are summarized in Table S1 and do not differ from the other patients from whom successful cultures were obtained.

**Figure 2:**
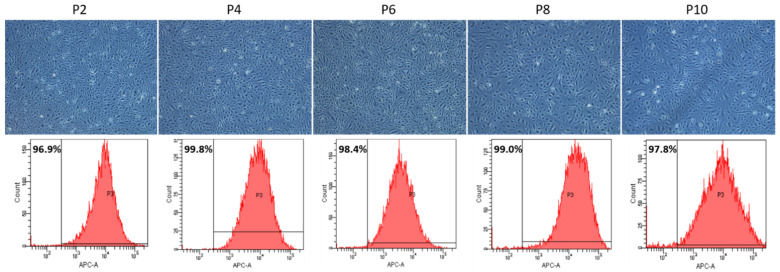
Representative pictures of PAECs showing shape, structure and cobblestone aspect of PAECs throughout subcultures (**upper panel**). Quantitative analysis of CD31-positive PAECs by flow cytometry throughout subcultures (**lower panel**). Scale = 200 µm.

PAECs freshly isolated from *Swan-Ganz* pulmonary arterial catheters displayed a typical cobblestoned morphology (Figure 2; Subculture 2, S2). Until Subculture 6, we did not observe any major changes in the morphology of PAECs (Figure 2, S2–S6). By contrast, after eight subcultures, cell hypertrophy and occurrence of myofibroblast-like cells could be observed, which was further enhanced after 10 subcultures (Figure 2, S8–S10). Concomitantly, the CD31-positive PAECs measured by flow cytometry showed the presence of homogenous endothelial cell populations over subcultures (Figure 2). However, the spreading of CD31-positive cells, shown by the enlargement of the base of the curve, indicated a higher variety of CD31 expression or physical properties of the cells (Figure 2).

**Figure 3:**
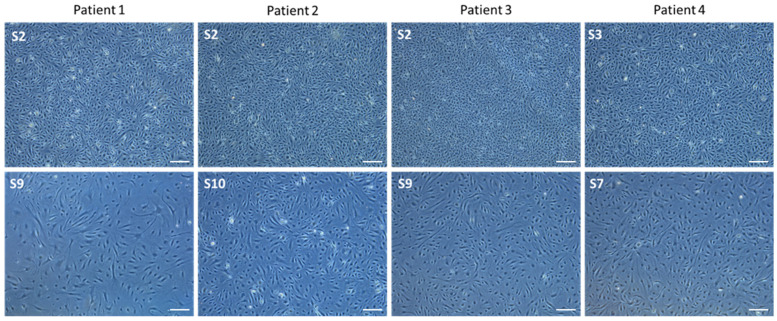
Representative pictures of PAECs in culture isolated from 4 different patients at right heart catheterization using *Swan-Ganz* pulmonary arterial catheters. (**upper panel**): early subcultures; (**lower panel**): advanced subcultures. Scale = 200 µm.

In early subcultures (S2 and S3), a typical cobblestoned morphology of PAECs isolated from four different patients was observed. In advanced subcultures (S7–S10), elongated myofibroblast-like and hypertrophic cells occurred (Figure 3).

**Figure 4:**
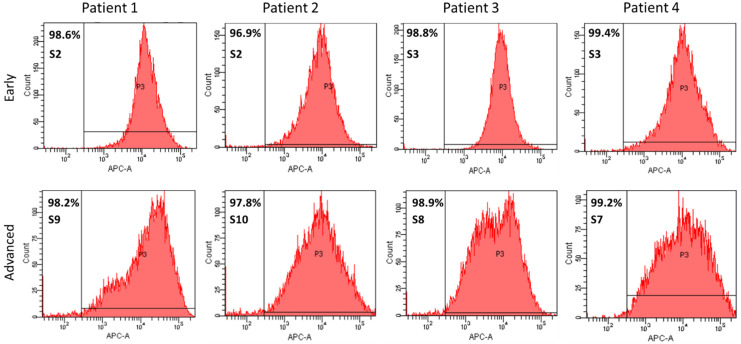
Quantitative phenotyping by flow cytometry of CD31-APC-labeled PAECs isolated from 4 different patients using *Swan-Ganz* pulmonary arterial catheters. Histograms at early (**upper panel**) and advanced (**lower panel**) subcultures.

In early subcultures (S2 and S3), a highly homogenous endothelial cell population was observed, as shown by the percentage of 98.7 ± 1.1% of CD31-positive cells detected by flow cytometry (Figure 4). Interestingly, in advanced subcultures (S8–S10), the percentage of CD31-positive cells remained unchanged 98.4 ± 0.6%: *p* = 0.404), despite the enlargement of the histogram width (Figure 4).

### 3.3. Qualitative Phenotyping of PAECs Using Endothelial-Specific Markers in Early and Late Subcultures

**Figure 5:**
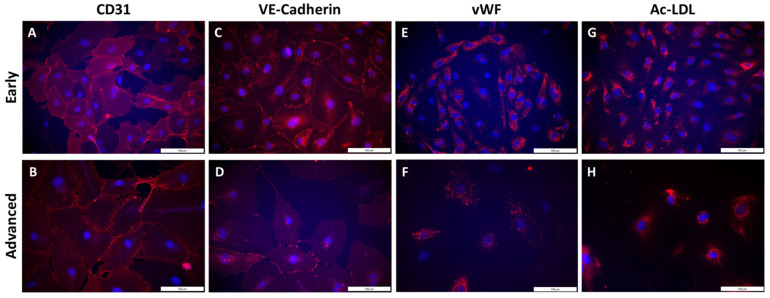
Qualitative phenotyping of PAECs using endothelial-specific markers in early and advanced subcultures. Staining of endothelial surface markers CD31 (**A**,**B**) and VE-Cadherin (**C**,**D**), endothelial intracellular markers von Willebrand factor (vWF; **E**,**F**) and Ac-LDL uptake (**G**,**H**) was performed by immunofluorescence in early (**A**,**C**,**E**,**G**) and advanced subcultures (**B**,**D**,**F**,**H**). Nuclei were counterstained using DAPI (blue). Scale = 100 µm.

**Figure 6:**
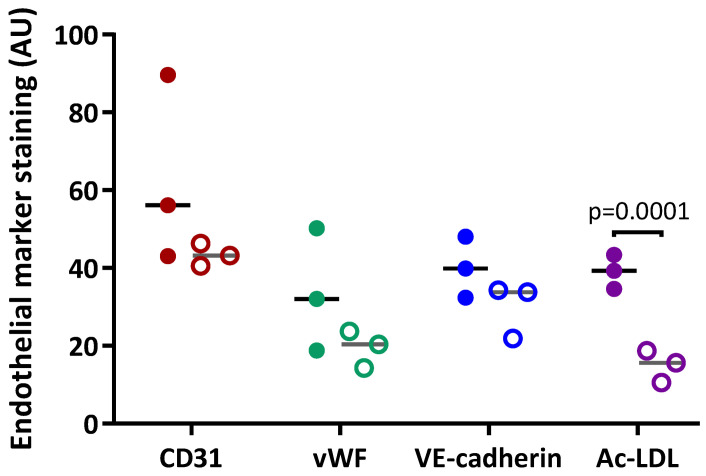
Quantification of the expression of specific endothelial markers in PAECs. Expression of CD31, vascular endothelium cadherin (VE-cadherin) and von Willebrand factor (vWF)and uptake of acetylated low-density lipoproteins (Ac-LDL) in PAECs from 3 different patients were quantified in early subcultures (**full circles**) and advanced subcultures (**open circles**).

In early subcultures (S2 to S4), isolated PAECs showed expression of the specific endothelial cell surface markers CD31 (Figure 5A) and VE-Cadherin (Figure 5C). In advanced subcultures (S7 to S10), expression of both CD31 (Figure 5B) and VE-Cadherin (Figure 5D) was mitigated. VE-cadherin staining was less homogenous with visual gaps (Figure 5D). Quantification of the immunofluorescence did not show any significant change in either CD31 or VE-cadherin staining between early and advanced subcultures (Figure 6).

**Figure 7:**
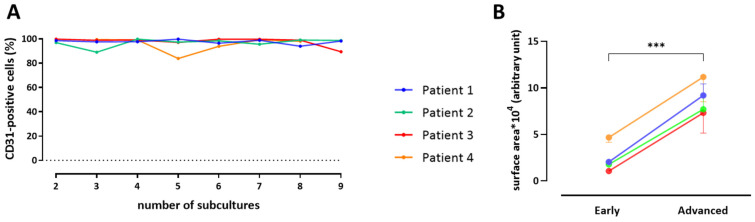
CD31-positive cells isolated from 4 individual patients throughout subcultures (**A**). Quantitative analysis of endothelial cell hypertrophy from 4 individual patients at early and advanced subcultures (**B**). Paired *t*-test, *** *p* = 0.0001.

Cell hypertrophy was observed (Figure 5B,D), with a significant increase in cell surface area in advanced compared to early subcultures (Figure 7B). Intracellular expression of the specific endothelial marker vWF (Figure 5E) and the uptake of Ac-LDL by endothelial-specific scavenger receptors (Figure 5G) were profuse in early subcultures and seemed to be reduced in advanced subcultures (Figure 5F,H), with a significant decrease in Ac-LDL uptake in advanced compared to early subcultures (Figure 6).

Additionally, early subcultures of PAECs did not display any positive staining for α-SMA (Figure S1); elongated myofibroblast-like cells observed in advanced subcultures (Figure 3) did not harbor αSMA-stained fibers (Figure S1). Finally, the number of CD31-positive cells, quantified by flow cytometry, remained stable throughout subcultures (Figure 7A). 

## 4. Discussion

In the present study, we reported a detailed and fine-tuned protocol allowing for the isolation of PAECs from *Swan-Ganz* pulmonary arterial catheters, adapted from the initial description of the technique by Pollett et al. [9], although different from the more recently described methodology [10]. More importantly, we demonstrated that PAECs harvested from *Swan-Ganz* pulmonary arterial catheters can be maintained in vitro, with an endothelial phenotype remaining stable for several weeks in culture. 

A major finding of the present study was the possibility to maintain PAECs harvested from pulmonary arterial catheters in culture, with a rather stable phenotype throughout subcultures. Whereas the pioneers who initiated the technique settled for cell isolation, we and Ventetuolo et al. [10] proceeded a step further by maintaining and expanding cells in culture. As initially reported [9], we obtained homogenous cultures of cobblestoned ECs, independently of disease etiology. Consequently, we did not perform extensive characterization of the isolated cell populations by flow cytometry because potential contaminations by other cell types would be wiped out by the stringent EC culture conditions. In more advanced subcultures (>S6), loss of endothelial markers and cell hypertrophy indicate cell dedifferentiation, as observed in human primary lung endothelial cells in culture. This suggests that short-term subcultures would be more appropriate for potential translational applications, in agreement with previous findings highlighting the in vitro retention of several abnormal phenotypic features in cultured pulmonary endothelial cells from patients with PAH [13]. 

To build upon the technique developed by Pollett [9] and Passineau [14], we introduced the following amendments: (i) immediate immersion of the catheter in warmed basal medium containing 0.2% cell growth supplement instead of iced collection; (ii) inflated balloon agitated in cell culture medium; (iii) omission of the micro-bead purification step, which might lower cell recovery yield [14]; (iv) unbroaching of cells for the first 5 days in culture instead of refreshing medium the day after isolation (see Table S2). Into our hands, the current protocol resulted in the successful isolation of PAECs followed by subcultures from about 30 *Swan-Ganz* pulmonary arterial catheters within 5 years. This indicates that, by contrast to PAECs isolated from lung tissue at transplantation from patients with advanced stage of the disease, the present technique is a unique and inestimable source of PAECs at diagnosis and follow-up, enabling serial collection along disease progression and/or in response to treatment without additional risk to the patient. Using our protocol described above, we achieved a success rate of 35%, which is similar to the success rate described by Ventetuolo et al. [10], although the isolation technique is slightly different, indicating that this procedure can be replicated to expand the use of this technique and increase the number of patient-specific PAECs. 

A major benefit of this protocol is the ability to exclusively harvest endothelial cells originated from the pulmonary vessel wall, excluding circulating endothelial cells, as previously argued [9]. This was confirmed by Ventetuolo et al., who showed that PAECs express endothelial cell markers similarly to commercially available human PAECs and observed that the balloon of the *Swan-Ganz* pulmonary arterial catheter has to be inflated within the pulmonary artery with physical contact between the balloon and the pulmonary vascular wall to be able to collect PAECs. [10]. In addition, molecular signature may be possible as recently proposed for in situ expression of Bcl-2 in PAECs from patients with pulmonary hypertension relative to heart failure with preserved ejection fraction [15]. 

However, such a technique displays some limitations that need to be highlighted. By contrast to isolation of endothelial cells from peripheral blood, e.g., BOECs [16] or iPSCs [8], the present technique does not offer the possibility to sample healthy controls or unaffected *bmpr2* mutation carriers. Nevertheless, synergy between these different methods will increase the insights of endothelial cell biology during disease progression, disease heterogeneity and specific response to treatment. The rather low and highly variable number of viable cells harvested obviously conditioned the success rate, which may depend on patient etiology, technique of the physician and period during which the balloon is in contact with the vessel wall. This location is generally the branch of a pulmonary artery, and not the pre-capillary/microvessels initially affected by the disease. Impaired functionality of PAECs from lobar and/or (sub)segmental pulmonary arteries may not fully represent those of pre-capillary pulmonary vessels involved in PAH; however, Ventetuolo et al. [10] interestingly highlighted the rationale for studying proximal PAECs, since wall stress changes in proximal pulmonary arteries contribute to compliance and coupling in PAH. Moreover, the genetic background is the same in macrovascular and microvascular pulmonary ECs, which is an important aspect to be considered in precision medicine.

## 5. Conclusions

To conclude, the present study highlights and confirms the promising and unique opportunity to obtain homogenous and rather stable subcultures of primary PAECs from a larger cohort of patients, more frequently, at diagnosis and follow-up. Within the era of precision medicine, including alternative and innovative therapeutic approaches combining gene editing, cell therapy and oral medication [17], we believe that this methodology will undeniably open multiple translational and clinical applications and offer promising perspectives for patients suffering from rare pulmonary vascular diseases.

## Figures and Tables

**Table 1:** Patient characteristics.

	All (*n =* 132)	Successful (*n =* 56)	Unsuccessful (*n =* 76)	*p*-Value
**Age, years**	64 (23–89)	64 (27–89)	64 (23–83)	0.72
**Gender, male (%)**	33	37	30	
**BMI kg/m^2^**	25 (17–47)	24 (18–47)	26 (17–41)	0.54
**Etiology (%)**				
***IPAH***	*34*	*32*	*36*	
***HPAH***	*4*	*5*	*3*	
***Drug/toxin***	*6*	*7*	*5*	
***APAH***	*13*	*7*	*17*	
***PVOD***	*2*	*4*	*1*	
***PH sec to HD***	*5*	*2*	*8*	
***PAH sec to LD***	*2*	*4*	*1*	
***CTEPH***	*25*	*30*	*21*	
***No PH***	*8*	*9*	*8*	
**PAH-specific therapy (%)**				
***treatment-naive***	*49*	*45*	*53*	
***monotherapy***	*12*	*14*	*11*	
***dual therapy***	*21*	*20*	*22*	
***triple therapy***	*15*	*18*	*13*	
***CCB***	*2*	*4*	*1*	
**NYHA FC**				0.43
***I***	*12*	*5*	*7*	
***II***	*61*	*25*	*36*	
***III***	*54*	*23*	*31*	
***IV***	*5*	*3*	*2*	
**RAP (mmHg)**	7 (1–25)	7 (2–25)	7 (1–22)	0.46
**mPAP (mmHg)**	41 (11–106)	39.5 (11–70)	43 (13–106)	0.75
**PAWP (mmHg)**	10 (2–57)	10 (4–57)	10 (2–22)	0.70
**PVR (dyn·s·sec^−5^)**	530 (61–2187)	539 (61–2187)	527 (72–1706)	0.97
**CI (L/min/m^2^)**	2.5 (1.1–4.7)	2.5 (1.1–3.9)	2.6 (1.3–4.7)	0.33
**SvO_2_ (%)**	65.5 (37–95)	65 (39–80)	66 (37–95)	0.38
**6–MWD (m)**	360 (23–747)	386 (60–580)	349 (23–747)	0.66
**NT–proBNP (ng/mL)**	807 (8–21834)	753 (53–21834)	807 (8–13395)	0.55

Cultures were considered successful when an expanding cell colony was observed; if not observed, these cultures were considered unsuccessful. Results are expressed as median (min-max). BMI (body mass index); IPAH (idiopathic pulmonary arterial hypertension); HPAH (heritable PAH); APAH (associated PAH (including associations with connective tissue disease (CTD), congenital heart defects (CHD), porto-PH (PoPH) and HIV)); PVOD (pulmonary veno-occlusive disease); PH sec to HD, pulmonary hypertension secondary to heart disease; PH sec to LD, PH secondary to lung disease; CCB (calcium channel blockers); RAP (right atrial pressure); mPAP (mean pulmonary arterial pressure); PAWP (pulmonary arterial wedge pressure); PVR (pulmonary vascular resistance); CI (cardiac index); SvO2 (mixed venous oxygen saturation); 6-MWD (six-minute walking distance); and NT-proBNP (N-Terminal-pro-brain natriuretic peptide) were recorded.

**Table 2:** Summary of total collected catheters and successfully stored PAECs.

	2015	2016	2017	2018	2019	Total
**Total SG catheters collected**	15	17	34	43	23	132
**Successful culture (%)**	6 (40%)	4 (24%)	15 (44%)	20 (47%)	11 (48%)	56 (42%)
**Stored PAECs (%)**	4 (25%)	3 (18%)	2 (6%)	6 (14%)	8 (35%)	23 (27%)

SG, *Swan-Ganz* catheters; PAEC, pulmonary arterial endothelial cells.

## Data Availability

The data are available on request from the corresponding author.

## Supplementary Materials

The following are available online at https://www.mdpi.com/article/10.3390/cells10113229/s1, Table S1. Patient characteristics from whom cells were phenotyped (n = 4/56); Table S2. Summary of catheter collection and cell harvest conditions according to different protocols; Figure S1. PAECs isolated from Swan-Ganz pulmonary catheters in early and advanced subcultures, stained with antibodies against αSMA.
