# Supplementary material for: Incremental Experience in In Vitro Primary Culture of Human Pulmonary Arterial Endothelial Cells Harvested from Swan-Ganz Pulmonary Arterial Catheters
Source: Cells. 2021 Nov 19;10(11):3229. doi: 10.3390/cells10113229 (PMC8618201; doi:10.3390/cells10113229)
Supplement: Supplementary file 1 [file cells-10-03229-s001.zip › cells-1352064-supplementary.pdf]

cells

MDPI

Article

Incremental experience in *in vitro* primary culture of human pulmonary arterial endothelial cells harvested from *Swan-Ganz* pulmonary arterial catheters

Birger Tielemans[1], Leanda Stoian[2], Allard Wagenaar[2], Mathias Leys[3], Catharina Belge[4], Marion Delcroix[4] and Rozenn Quarck[4, *]

[1] Laboratory of Respiratory Diseases & Thoracic Surgery (BREATHE), Department of Chronic Diseases & Metabolism (CHROMETA) & Biomedical MRI, Department of Imaging and Pathology, University of Leuven, Leuven, Belgium.
[2] Laboratory of Respiratory Diseases & Thoracic Surgery (BREATHE), Department of Chronic Diseases & Metabolism (CHROMETA), University of Leuven, Leuven, Belgium.
[3] Clinical Department of Respiratory Diseases, University Hospitals, University of Leuven, Leuven, Belgium.
[4] Laboratory of Respiratory Diseases & Thoracic Surgery (BREATHE), Department of Chronic Diseases & Metabolism (CHROMETA), University of Leuven, Clinical Department of Respiratory Diseases, University Hospitals, University of Leuven, Leuven, Belgium.

Supplemental Data

Table S1. Patient characteristics from whom cells were phenotyped (n=4/56).

	Successful (n=52)	Phenotyping (n=4)	p-value
**Age, years**	64 (27-89)	64 (46-81)	0.80
**Gender, male (%)**	37	25	
**BMI kg/m^2**	24 (18-47)	26 (24-44)	0.31
**Etiology (%)**			
IPAH	*14*	*25*	
HPAH	*2*	-	
Drug/toxin	*3*	*25*	
APAH	*3*	-	
PVOD	*2*	-	
PH sec to HD	*1*	-	
PAH sec to LD	*2*	-	
CTEPH	*13*	*50*	
No PH	*4*	-	
**PAH-specific therapy (%)**			
treatment-naive	*48*	*25*	
monotherapy	*15*	*25*	
dual therapy	*21*	*50*	
triple therapy	*19*	-	
CCB	*4*	-	
**NYHA FC**			0.63
I	5	-	
II	23	2	
III	22	1	
IV	2	1	
**RAP (mmHg)**	7 (2- 25)	11 (2-11)	0.86
**mPAP (mmHg)**	39.5 (11-70)	48 (32-51)	0.54
**PAWP (mmHg)**	10 (4-57)	7 (5-12)	0.24
**PVR (dyn.s.sec^{-5})**	539 (61-2187)	690 (330-906)	0.61
**CI (L/min/m^2)**	2.5 (1.1-3.9)	2.4 (1.6-2.9)	0.68
**SvO_2 (%)**	65 (39-80)	63 (50-73)	0.57
**6-MWD (m)**	386 (60-580)	324 (72-552)	0.71
**NT-proBNP (ng/mL)**	753 (53-21834)	2131 (403-3694)	0.36

Among successful cultures, 4 patients were randomly selected for extensive phenotyping. Results are expressed median (min-max). BMI, body mass index; IPAH, idiopathic pulmonary arterial hypertension; HPAH, heritable PAH; APAH, associated PAH (including associations with connective tissue disease (CTD), congenital heart defects (CHD), porto-PH (PoPH) and HIV); PVOD, pulmonary veno-occlusive disease; PH due to HD, pulmonary hypertension secondary to heart disease; PH due to LD, PH secondary to lung disease; CCB, calcium channel blockers; RAP, right atrial pressure; mPAP, mean pulmonary arterial pressure; PAWP, pulmonary arterial wedge pressure; PVR, pulmonary vascular resistance; CI, cardiac index; SvO_2, mixed venous oxygen saturation; 6-MWD, six-minute walking distance; NT-proBNP, N-Terminal-pro-brain natriuretic peptide.

Table S2. Summary of catheter collection and cell harvest conditions according to different protocols

		**Pollet *et al.*[1]**	**Passineau *et al.*[2]**	**Ventetuolo *et al.*[3]**	**Tielemans *et al.***
**Catheter collection**	wash/flush catheter	no	sterile saline	no	no
	collection medium	endothelial cell media	EC growth medium	Media + EndoGRO (*Milipore Sigma)*	EC growth media (*Cell Applications)*
	temperature	4 °C	4 °C	37 °C	37 °C
	swirling	no	yes	no	yes
	temperature	undefined	4 °C	37 °C	37 °C
	balloon inflation	no	200µL medium	no	yes - air
**Cell harvest**	scraping of the balloon	no	yes	no	no
	trypsin solution	0.5% trypsin in PBS	cell detachment solution	no	Trypsin-EDTA
	centrifugation	10' at 1500 rpm	10' at 650g	no	10' at 500g
	RBC lysis buffer	yes – ACK-PBS solution	yes	no	yes - ACK lysis buffer
	use of microbeads	no	anti -CD146	no	no
	final centrifugation	10' at 2500 rpm	10' at 500 g	no	10' at 500 g
	use	cell sorting	fixation, staining, culture	catheter in culture	cells (pellet) in culture
**Refreshing medium**		Day after isolation	NA	Day after isolation	4 days post-isolation

References

1. Pollett JB, Benza RL, Murali S, Shields KJ, Passineau MJ. Harvest of pulmonary artery endothelial cells from patients undergoing right heart catheterization. J Heart Lung Transpl. 2013; 32: 746–749.
2. Passineau MJ, Gallo PH, Williams G, Perez R, Benza RL. Harvest of Endothelial Cells from the Balloon Tips of Swan-Ganz Catheters after Right Heart Catheterization. J. Vis. Exp. 2019; 143: e58353.
3. Ventetuolo CE, Aliotta JM, Braza J, Chichger H, Dooner M, McGuirl D, Mullin CJ, Newton J, Pereira M, Princiotto A, Quesenberry PJ, Walsh T, Whittenhall M, Klinger JR, Harrington EO. Culture of Pulmonary Arterial Endothelial Cells from Pulmonary Artery Catheter Balloon Tips: Considerations for Use in Pulmonary Vascular Disease. Eur. Respir. J. 2020; 1901313.

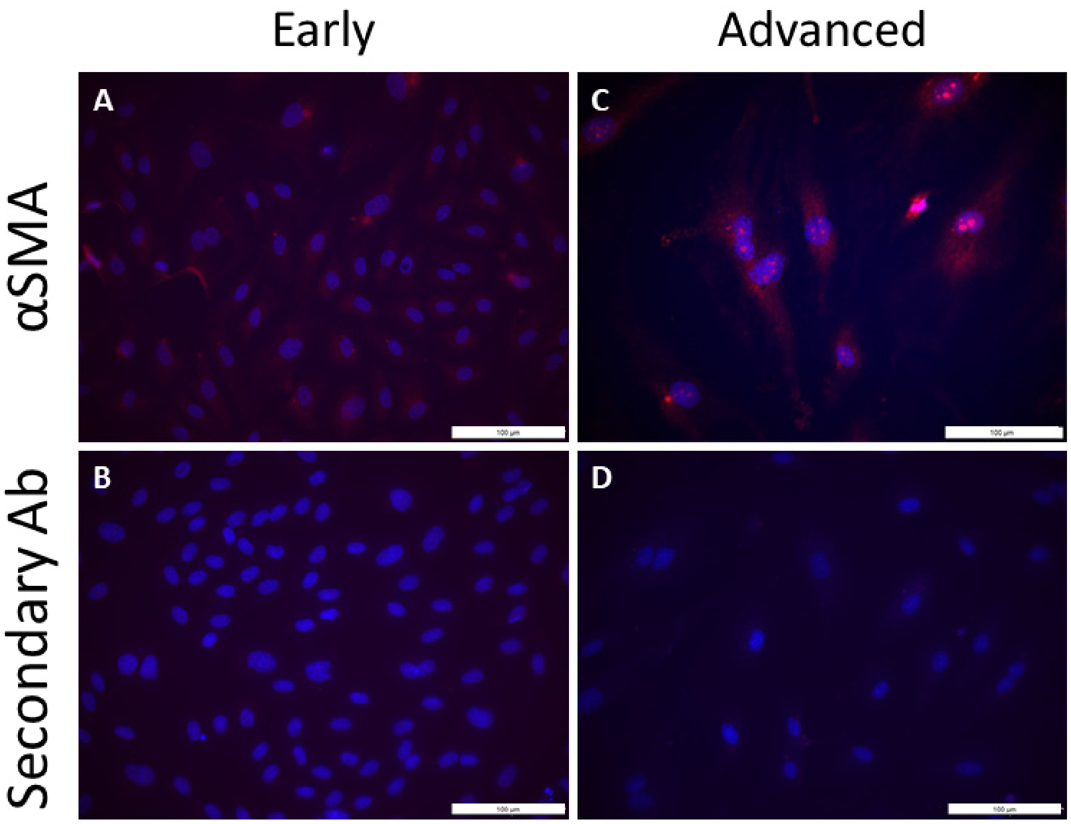

Figure S1. PAECs isolated from Swan-Ganz pulmonary catheters in early (**A, B**) and advanced subcultures (**C, D**), stained with antibodies against αSMA (**A, C**) and in absence of the primary antibody (**B, D**). Nuclei were counterstained using DAPI (blue). Scale = 100 μm.
